# A new species of the brown lacewing genus *Zachobiella* Banks from China (Neuroptera, Hemerobiidae) with a key to species

**DOI:** 10.3897/zookeys.502.9251

**Published:** 2015-05-04

**Authors:** Yang Zhao, Bingzhen Yan, Zhiqi Liu

**Affiliations:** 1 Department of Entomology, China Agricultural University, No. 2 Yuanmingyuan West Road, Beijing 100193, China

**Keywords:** Notiobiellinae, *Zachobiella*, China

## Abstract

The genus *Zachobiella* Banks, 1920 is reviewed and a new species *Zachobiella yunanica* **sp. n.** described from China. All species found in China are redescribed, and *Zachobiella submarginata* Esben-Petersen, 1929 is recorded from China for the first time. A key to the adults of *Zachobiella* is provided.

## Introduction

The genus *Zachobiella* was erected by Banks (1920) based on the type species *Zachobiella punctata*. This genus belongs to the subfamily Notiobiellinae (Nakahara 1960) and is distributed throughout southeast Asia and Australia. It is diagnosed by the following forewing characteristics: anterior radial trace bearing two prestigmal radial sectors, all posthumeral costal veinlets simple and trichosores not evident in the humeral area; the males also typically have highly ornate genitalic armature (Oswald 1993).

Banks (1939) described *Zachobiella hainanensis* from China based on single specimen collected from Hainan province; Nakahara (1966) subsequently described the species *Zachobiella striata* from Taiwan. Presently, nine species are described world-wide (New 1988a, 1988b; Oswald 1993, 2014) with four species recorded from China, including the new species described herein from China.

In this paper, all the known species of genus *Zachobiella* in China are redescribed and illustrated, including detailed descriptions and illustrations of the new species *Zachobiella yunanica* sp. n.; *Zachobiella submarginata* Esben-Petersen is recorded from China for the first time. In addition, a key for identification of adults is also presented. All specimens are deposited in the Entomological Museum of China Agricultural University (CAU), Beijing.

## Material and methods

Specimens were examined under an Optec SZ760 stereomicroscope. Images of wings were taken with a Nikon EOS D3200 digital camera attached to the stereomicroscope. The terminalia were observed under a Leica DM2500 compound microscope. Descriptions of colouration are based on observations under the stereomicroscope with direct light on specimens preserved in 75% ethyl alcohol. The abdominal apex with genitalia was cut off and heated in 10% sodium hydroxide for about 10–20 min and then transferred to an excavated slide with glycerin. After examination it was transferred to fresh 75% ethyl alcohol and stored in a microvial.

Wing venation terminology follows Oswald (1993) and Makarkin and Wedmann (2009). Terminology of genitalia follows Oswald (1993).

Abbreviations: 7s 7^th^ sternite; 8s 8^th^ sternite; 9s 9^th^ sternite; 7t 7^th^ tergite; 8t 8^th^ tergite; 9t 9^th^ tergite; ect ectoproct; ehgs extrahemigonarcus; hgs hemigonarcus; ihgs intrahemigonarcus; med mediuncus; orb# oblique radial branch of anterior radial trace (= radial sector).

## Taxonomy

### Key to species of *Zachobiella*

**Table:** 

1	Small triangular dark spots present at the forks of longitudinal veins in forewing	**2**
–	Small triangular dark spots absent at the forks of longitudinal veins in forewing	**6**
2	Only one crossvein present in hind wing	**3**
–	Two crossveins present in hind wing	**5**
3	3ir1 located before the fork of orb2 in forewing	***Zachobiella submarginata* Esben-Petersen**
–	3ir1 located after the fork of orb2 in forewing	**4**
4	Two gradate series present in forewing; male genitalia with both posterodorsal and posteroventral edges of ectoproct extending upwards into long arms	***Zachobiella yunanica* sp. n.**
–	Three gradate series present in forewing; male genitalia with only posteroventral edge of ectoproct extending upwards into long arm	***Zachobiella lobata* New**
5	Rs forked at the base in hind wing	***Zachobiella punctata* Banks**
–	Rs forked in the middle in hind wing	***Zachobiella marmorata* Navás**
6	3ir1 located before the fork of orb2 in forewing	**7**
–	3ir1 located after the fork of orb2 in forewing	**8**
7	Approximately 12 segments of distal flagellum obviously darker than the others in antennae; obvious brown stripes present in forewing	***Zachobiella striata* Nakahara**
–	Basal half of antennae obviously darker than the others; obvious brown stripes absent in forewing	***Zachobiella jacobsoni* Esben-Petersen**
8	Two crossveins present in hind wing; pronotum brown while paler on sides	***Zachobiella pallida* Banks**
–	Only one crossvein present in hind wing; pronotum yellowish-brown while brown longitudinal stripes present along both sides	***Zachobiella hainanensis* Banks**

### Zachobiella yunanica

Taxon classificationAnimaliaNeuropteraHemerobiidae

Zhao, Yan & Liu sp. n.

http://zoobank.org/7188F319-D5E8-4899-9213-26300007A24A

Figs 1 , 5–8

#### Diagnosis.

Triangular dark spots present at the forks of longitudinal veins in forewing and 3ir1 present after the fork of orb2. Male: posterodorsal edge of 7^th^ tergite slightly extending backwards; a pair of rough spiny projections present on the dorsal surface of the 8^th^ tergite; posteroventral edge of the 9^th^ tergite extending upwards with serrated inner margin from one third distally; both posterodorsal and posteroventral edges of ectoproct extending upwards into long arms, especially the posteroventral.

#### Measurements.

Forewing length 5.4–6.0 mm, width 2.1–2.2 mm. Hind wing length 4.4–4.7 mm, width 1.7–1.9 mm. Body length 4.0–5.3 mm.

#### Description.

*Head.* Yellowish-brown. Brown stripe present from the rear of eye to the mandible. Labial and maxillary palpi brown. Antenna light brown, more than fifty segments. Eyes black with a metallic luster.

*Thorax.* Yellowish-brown, with brown longitudinal stripes along both sides of tergites. Legs yellowish-brown with no spots.

**Figures 1–4.**
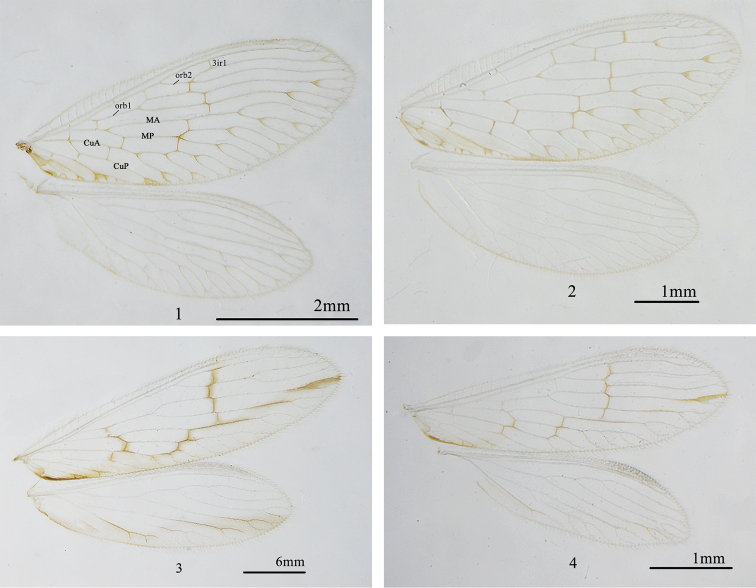
Wings. **1** *Zachobiella yunanica* Zhao, Yan & Liu, sp. n. **2** *Zachobiella submarginata* Esben-Petersen, 1929 **3** *Zachobiella striata* Nakahara, 1966 **4** *Zachobiella hainanensis* Banks, 1939.

*Wings* (Fig. 1). Forewing shape oval. Yellowish-brown and hyaline; triangular dark spots present at the forks of longitudinal veins; veins yellowish-brown with crossveins brown. Anterior radial trace bearing two ORB’s, with two secondary branches respectively; 3ir1 present after the fork of orb2; 3ir2 present after the fork of orb1 and before the fork of orb2. M with two branches, MA forked into 2–3 branches after the gradate series and MP into four branches. CuA with four branches. CuP simple. Two gradate series, inner gradate series with three crossveins and the outer with six. Hind wing oval. Pale yellow, hyaline; immaculate; veins pale yellowish-brown. Rs forked at base with four branches. M forked into two branches, with two secondary branches respectively after the gradate series. CuA with 3–4 branches. CuP simple. One gradate series, with only one crossvein r-m.

**Figures 5–8.**
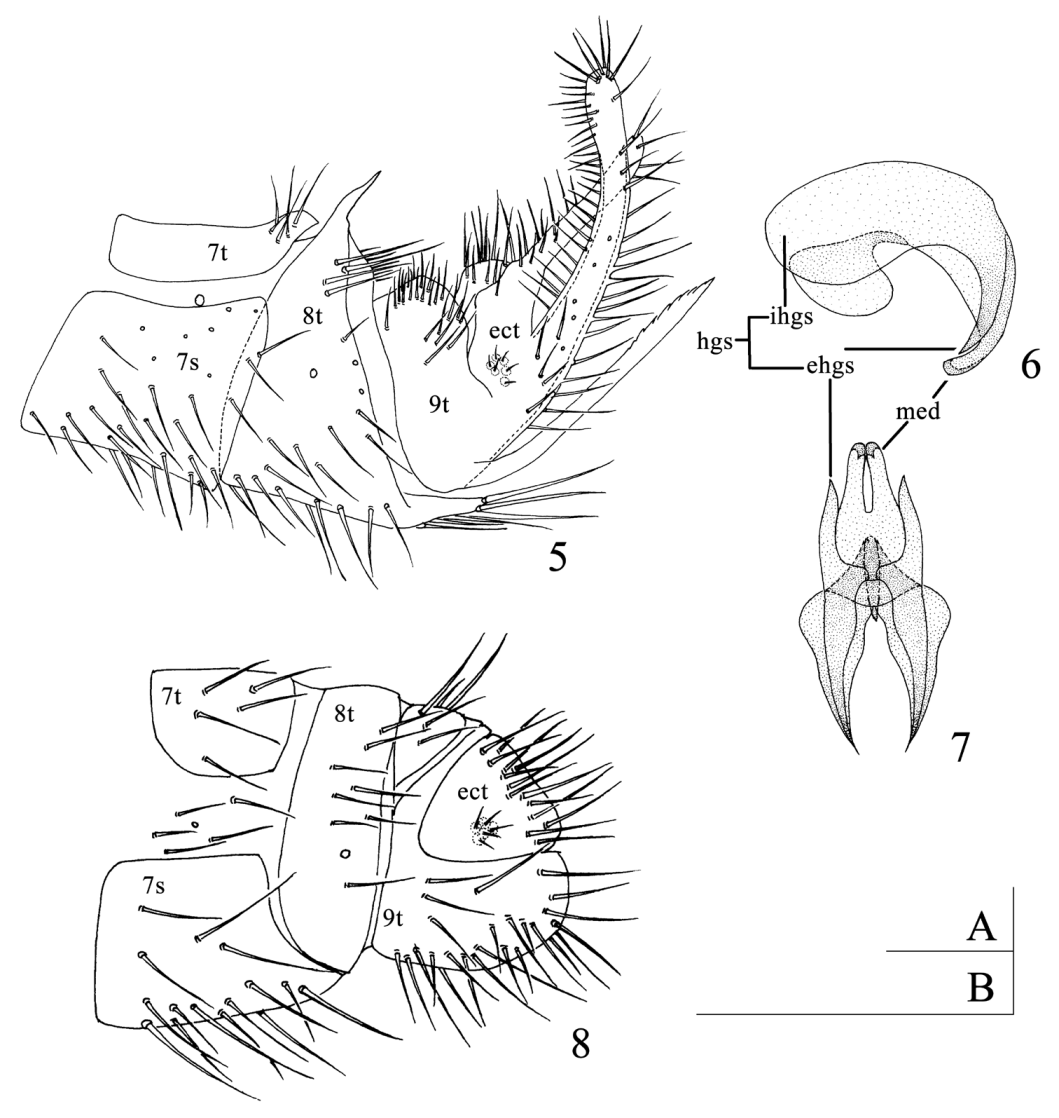
*Zachobiella yunanica* Zhao, Yan & Liu, sp. n. **5** Male terminalia, lateral view (B) **6** Gonarcus, lateral view (A) **7** Gonarcus, dorsal view (A) **8** Female terminalia, lateral view (B) Scale bars: 0.1 mm (A); 0.5 mm (B).

*Abdomen.* Yellowish-brown. Pilose. *Male terminalia* (Fig. 5). Posterodorsal edge of 7^th^ tergite slightly extending backwards, with a group of setae on the surface. 8^th^ tergite fused with the 8^th^ sternite, with a pair of distinct rough spiny projections dorsally, stout bristle tufts present below in lateral view; posteroventral edge extending backwards as a stout spine, densely covered with long setae. 9^th^ tergite covered with short setae on the dorsal and posteroventral edge extending upwards, blade-shaped, with serrated inner margin from one third distally. The dorsal surface of ectoproct protruding slightly and densely covered with short setae; both posterodorsal and posteroventral edges extending upwards into long arms, densely covered with setae. Mediuncus of gonarcus (Figs 6–7) consisting of a pair of long curved hooks, smooth surface without any spines; extrahemigonarcus long and tapering distally as a stout spine; hemigonarcus connected into a bridge internally. *Female terminalia* (Fig. 8). 9^th^ tergite split into two parts, the hind margin in the ventral part exceeding the posterior of ectoproct slightly. Ectoproct subtriangular in lateral view. Subgenitale absent.

#### Distribution.

China (Yunnan).

#### Material examined.

CHINA: Holotype, 1♂, Yunnan province, Ruili city, Mengxiu county, Gaoerxing. 4.v.1981, Chikun Yang (CAU). Paratypes, 1♂, Yunnan province, Ruili city, Mengxiu county, Gaoerxing. 5.v.1981, Chikun Yang (CAU); 1♀, Yunnan province, Ruili city, Mengxiu county, Gaoerxing. 2.v.1981, Chikun Yang (CAU).

#### Etymology.

The specific name refers to the type locality where this species is found.

#### Remarks.

This new species is closely related to *Zachobiella lobata* New, 1988, *Zachobiella punctata* Banks, 1920, and *Zachobiella submarginata* Esben-Petersen, 1929 based on the small triangular dark spots present at the forks of longitudinal veins in forewing. It can be distinguished from *Zachobiella submarginata* by 3ir1 present after the fork of orb2 in forewing while in *Zachobiella submarginata* it present before the fork of orb2. It also can be easily distinguished from *Zachobiella punctata* by having only one crossvein of gradate series in hind wing while in *Zachobiella punctata* there are two crossveins. In this new species two gradate series are present in the forewing and both the posterodorsal edge and the posteroventral edge of the ectoproct extend upwards into long arms. In *Zachobiella lobata* three gradate series are present in the forewing and only the posteroventral edge of the ectoproct extends upwards.

### Zachobiella submarginata

Taxon classificationAnimaliaNeuropteraHemerobiidae

Esben-Petersen

Figs 2 , 9

Zachobiella submarginata Esben-Petersen, 1929: 33.

#### Diagnosis.

Triangular dark spots present at the forks of longitudinal veins in forewing and 3ir1 present before the fork of orb2; Rs forked at the base in hind wing. Female: hind margin of 9^th^ tergite depressed forwards from lateral view and the hind margin of ventral part exceeding the posterior of ectoproct.

#### Measurements.

Forewing length 5.5–5.9 mm, width 1.9–2.2 mm. Hind wing length 4.0–4.8 mm, width 1.5–1.8 mm. Body length 4.2–5.0 mm.

#### Description.

*Head.* Yellowish-brown. Brown stripe present from the rear of eye to the mandible. Labial and maxillary palpi brown. Antenna light brown, more than fifty-five segments. Eyes black with a metallic luster.

*Thorax.* Yellowish-brown, with brown longitudinal stripes along both sides of tergite. Legs yellowish-brown without spots.

*Wings* (Fig. 2). Forewing oval. Yellowish-brown and hyaline, a pale brown stripe present from the base, along the hind margin and small triangular dark spots present at the forks of longitudinal veins; veins yellowish-brown with crossveins brown. Anterior radial trace bearing two ORB’s, with two secondary branches respectively; 3ir1 present before the fork of orb2; 3ir2 present after the fork of orb1 and before the fork of orb2. M with two branches and with two secondary branches respectively. CuA with four branches. CuP simple. Two gradate series, inner gradate series with two crossveins and the outer with six. Hind wing oval, pale yellow, hyaline; immaculate; veins pale yellow. Rs forked at base with four branches. M forked into two branches, with two secondary branches respectively after the gradate series. CuA with 3–4 branches. CuP simple. One gradate series, with the only one crossvein r-m.

**Figures 9–10.**
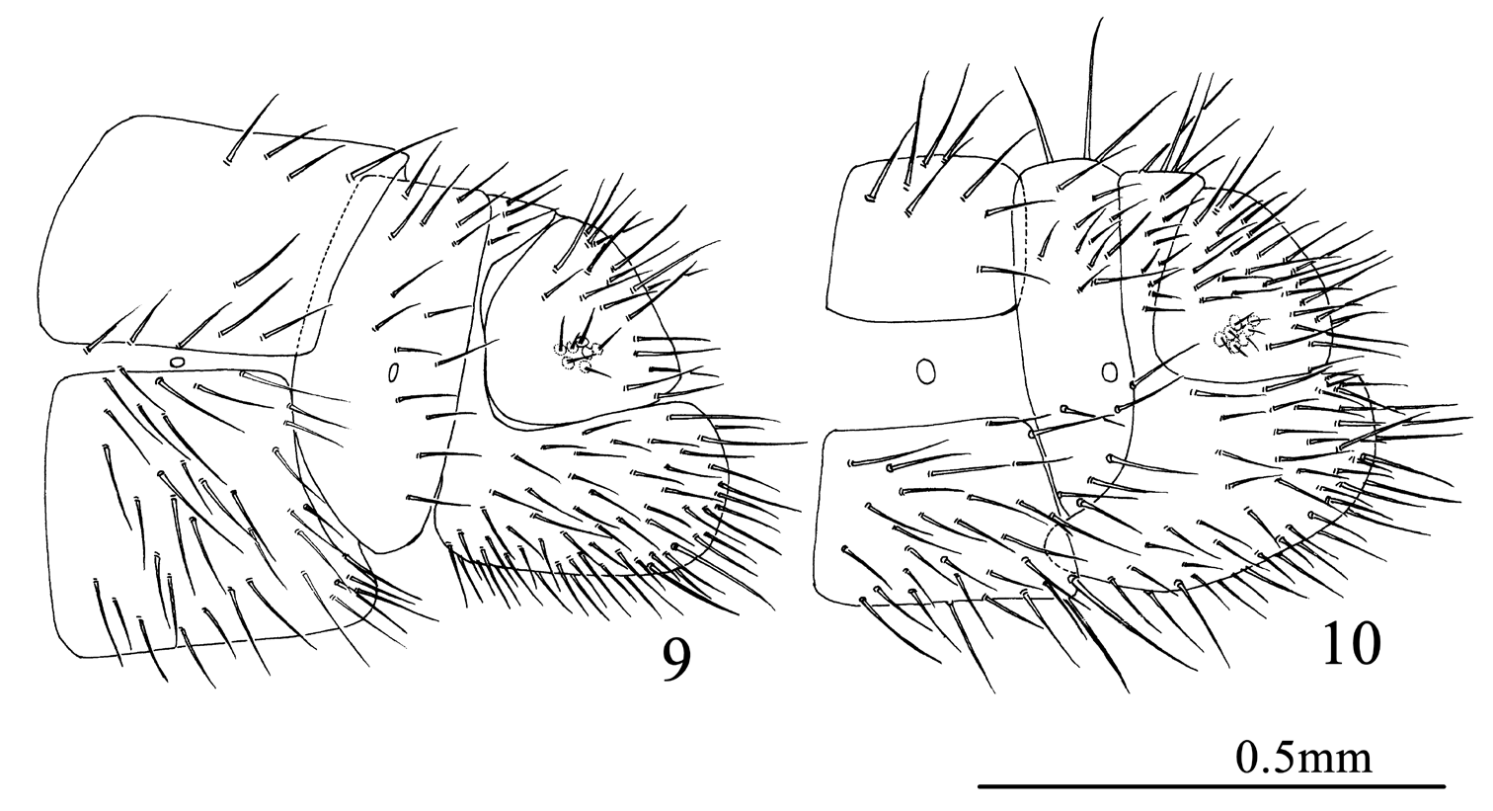
**9** *Zachobiella submarginata* Esben-Petersen, 1929. Female terminalia, lateral view **10** *Zachobiella striata* Nakahara, 1966. Female terminalia, lateral view. Scale bar: 0.5 mm.

*Abdomen.* Yellowish-brown. Hairy. *Female terminalia* (Fig. 9). Hind margin of 9^th^ tergite depressed forwards, hind margin in the ventral part exceeding the posterior of ectoproct. Ectoproct subtriangular from lateral view. Subgenitale absent.

#### Distribution.

China (Yunnan, Guangxi)

#### Material examined.

CHINA: 3♀, Yunnan province, Ruili city, Mengxiu county. 2.v.1981, Chikun Yang (CAU); 4♀, Yunnan province, Ruili city, Mengxiu county, Gaoerxing. 4.v.1981, Chikun Yang (CAU); 1♀, Yunnan province, Ruili city, Mengxiu county, Gaoerxing. 4.v.1981, Fasheng Li (CAU); 1♀, Yunnan province, Puer city, Lancang county. 20.iv.1981, Chikun Yang (CAU); 1♀, Guangxi province, Yulin city, Bobai county, Langping. 28.v.1982, Chikun Yang (CAU).

#### Remarks.

This species was described by Esben-Petersen (1929) from Australia without the description of genitalia; New (1988a) figured the male and female terminalia in his revision of the Australian brown lacewings. In this paper we figure the female genitalia and provide new distribution records for this species in China. This species is related to *Zachobiella yunanica* and *Zachobiella punctata* based on the triangular dark spots present at the forks of longitudinal veins in forewing. It can be distinguished from *Zachobiella punctata* by having only one crossvein of gradate series in the hind wing, while in *Zachobiella punctata* there are two crossveins.

### Zachobiella striata

Taxon classificationAnimaliaNeuropteraHemerobiidae

Nakahara

Figs 3 , 10

Zachobiella striata Nakahara, 1966: 198.

#### Diagnosis.

Forewing pale yellow and hyaline, thin brown stripe present along the gradate series and from the middle of MA to the lateral margin, an oval brown spot present at the margin, triangular dark spots absent at the forks of longitudinal veins and 3ir1 present before the fork of orb2. Female: 9^th^ tergite approximate ‘L’ -shaped from lateral view, anteroventral edge protruding forwards slightly.

#### Measurements.

Forewing length 4.5–5.7 mm, width 1.5–1.8 mm. Hind wing length 3.6–4.6 mm, width 1.2–1.5 mm. Body length 3.2–5.7 mm.

#### Description.

*Head.* Yellowish-brown, without any dark areas. Antenna more than sixty segments, most segments amber but over a dozen of segments of distal flagellum obviously darker than the others. Eyes black with a metallic luster. Mandibles brown.

*Thorax.* Yellowish-brown, with a light-coloured longitudinal stripe throughout. Legs yellowish-brown without spots; distal tarsomere darker than proximal four.

*Wings* (Fig. 3). Forewing narrow, apex slightly tapered. Pale yellow and hyaline, thin brown strips present along the gradate series and from the middle of MA to the lateral margin, an oval brown spot present at the margin; veins pale yellow and transparent with crossveins of gradate series brown. Anterior radial trace bearing two ORB’s, with two secondary branches respectively; 3ir1 present before the fork of orb2; 3ir2 present after the fork of orb1 and before the fork of orb2. M with two branches, MA forked into two branches after the gradate series and MP into 3–5 branches. Cu with two branches. Three gradate series, inner gradate series with 2–3 crossveins; middle with 2–3 and the outer with four. Hind wing narrow, apex slightly tapered. Pale yellow and hyaline, brown strip present from the base to the apex; veins yellow and transparent, crossvein of gradate series yellowish-brown. Rs forked in the middle with four branches. M forked into two branches, with 2–3 secondary branches respectively after the gradate series. Cu simple. One gradate series with the only one crossvein r-m.

*Abdomen.* Yellowish-brown, tergites and sternites brown, darker than the arthropleuron. Hairy. *Female terminalia* (Fig. 10). 9^th^ tergite approximate ‘L’-shaped from lateral view, anteroventral edge protruding forwards slightly, hind margin exceeding the hind margin of ectoproct. Ectoproct quadrant shaped from lateral view. Subgenitale absent.

#### Distribution.

China (Taiwan, Hainan, Yunnan)

#### Material examined.

CHINA: 2♀, Yunnan province, Dehong Autonomous Prefecture, Longchuan county. 5.v.1981, Chikun Yang (CAU); 1♀, Yunnan province, Hani-Yi Autonomous Prefecture of Honghe, Hekou town. 12.v.2011, Luxi Liu (CAU); 1♀, Hainan province, Wuzhishan city, Wuzhi hills. 16.v.2007, Yongjie Wang (CAU); 1♀, Taiwan province, Nantou city, Nantou County Council. 6.vii.2013, Xinyu Luo (CAU).

#### Remarks.

This species was described by Nakahara (1966) with only female specimens from Taiwan province and Iriomote Island without the description of genitalia. In this paper we describe the female, including the genitalia, and update the distribution records for this species in China. This species is similar to *Zachobiella pallida* Banks, 1939 and *Zachobiella jacobsoni* Esben-Petersen, 1926; however, it can be distinguished from *Zachobiella pallida* by 3ir1 present before the fork of orb2 in forewing while in *Zachobiella pallida* it is present after the fork of orb2. It also can be distinguished by the Rs forked in the middle in hind wing, while in *Zachobiella jacobsoni* the Rs is forked basally.

### Zachobiella hainanensis

Taxon classificationAnimaliaNeuropteraHemerobiidae

Banks

Figs 4 , 11–14

Zachobiella hainanensis Banks, 1939: 467.

#### Diagnosis.

Forewing pale yellow and hyaline, brown stripes present along the gradate series and from the middle of MA to the lateral margin, triangular dark spots absent at the forks of longitudinal veins and 3ir1 present after the fork of orb2. Male: dorsal surface of 8^th^ tergite protruding upwards as a short rod; dorsal of 9^th^ tergite slightly depressed and posteroventral edge extending upwards as a strong spine; dorsal of ectoproct obviously protruding upwards, posteroventral edge extending upwards into a thickened process. Female: 9^th^ tergite very small, triangular from lateral view; ectoproct large, slightly depressed in the middle of the hind margin.

#### Measurements.

Forewing length 3.2–4.9 mm, width 1.2–1.4 mm. Hind wing length 3.5–4.2 mm, width 1.1–1.3 mm. Body length 2.8–4.3 mm.

#### Description.

*Head.* Yellowish-brown, without any dark areas. Antenna amber, more than fifty-five segments, more than a dozen of segments of distal flagellum obviously paler than the others. Eyes black with a metallic luster.

*Thorax.* Yellowish-brown. Brown longitudinal stripes present along both sides of the pronotum; scutum of mesonotum and metanotum obviously brown. Legs yellowish-brown with no spots.

*Wings* (Fig. 4). Forewing narrow, apex slightly tapered. Pale yellow and hyaline, thin brown strips present along the gradate series and from the middle of MA to the lateral margin; veins pale yellow and transparent with crossveins of gradate series brown. Anterior radial trace bearing two ORB’s, with two secondary branches respectively; 3ir1 present after the fork of orb2; 3ir2 present after the forks of orb1 and orb2. M forked into two branches, MA simple, MP forked into 4–5 branches. Cu with two branches. Three gradate series, inner gradate series with 2–3 crossveins; middle with two and the outer with four. Hind wing narrow, apex slightly tapered. Pale yellow, hyaline and immaculate; veins pale yellow and transparent. Rs forked in the middle with four branches. M forked into two branches, with 2–3 secondary branches respectively after the gradate series. Cu simple. One gradate series, with the only one crossvein r-m.

**Figures 11–14.**
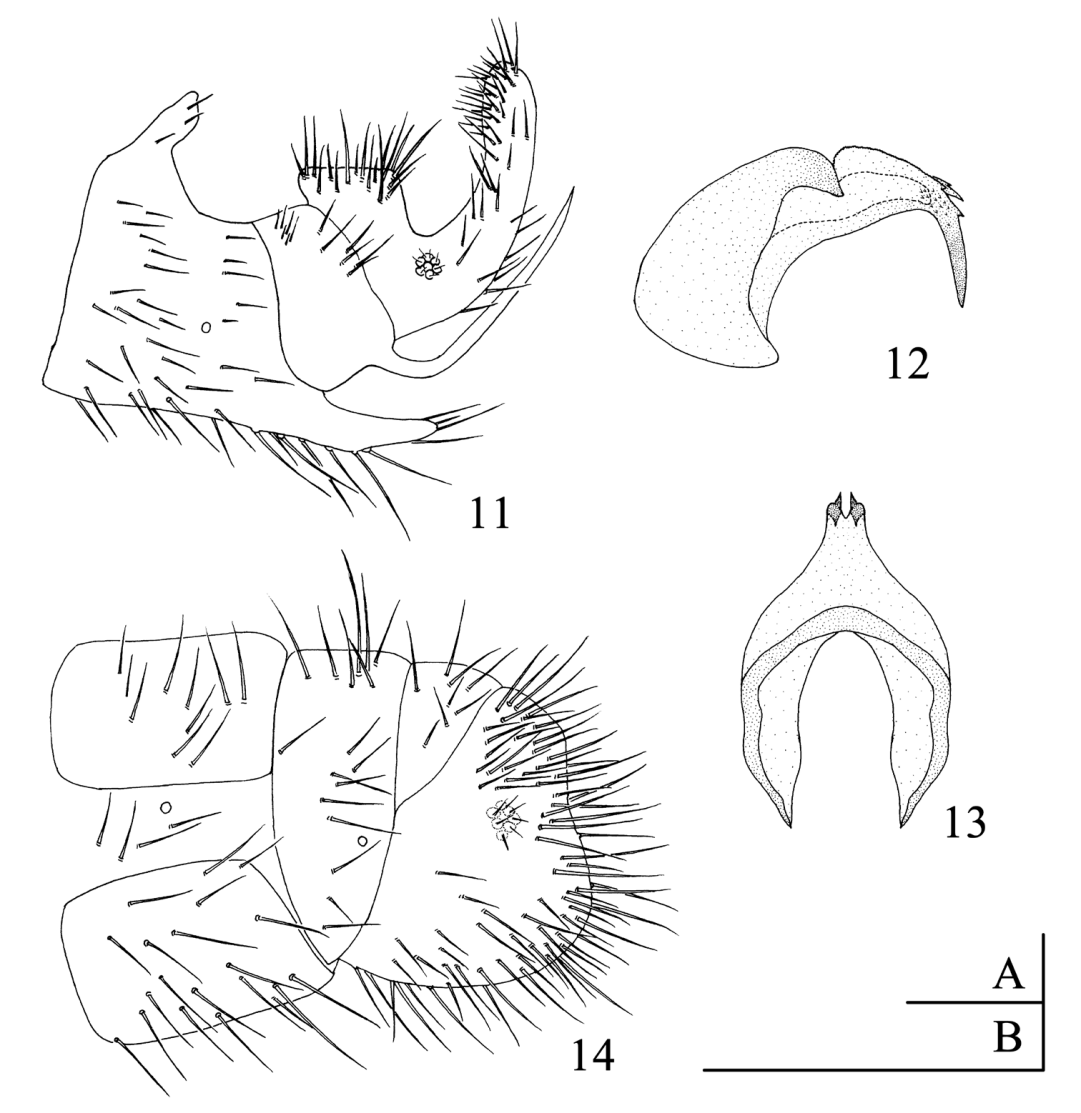
*Zachobiella hainanensis* Banks, 1939. **11** Male terminalia, lateral view (B) **12** Gonarcus, lateral view (A) **13** Gonarcus, dorsal view (A) **14** Female terminalia, lateral view (B). Scale bars: 0.1 mm (A); 0.5 mm (B).

*Abdomen.* Yellowish-brown, tergites and sternites brown, darker than the arthropleuron, pilose. *Male terminalia* (Fig. 11). 8^th^ tergite fused with the 8^th^ sternite, dorsal of both 8^th^ tergite protruding upwards as a short rod; posteroventral edge extending backwards as a stout spine, densely covered with long setae. Dorsal of 9^th^ tergite slightly depressed and posteroventral edge extending upwards as a strong spine. Dorsal surface of ectoproct obviously protruding upwards and densely covered with long setae; posteroventral edge extending upwards into a thickened process, densely covered with setae on the inner surface. Mediuncus of gonarcus (Fig. 12–13) consisting of a pair of long curved hooks, three to four stout spines present on the surface with small spines around; extrahemigonarcus small and slightly tapered. *Female terminalia* (Fig. 14). 9^th^ tergite very small, triangular from lateral view. Ectoproct large, slightly depressed in the middle of the hind margin. Subgenitale absent.

#### Distribution.

China (Hainan, Yunnan)

#### Material examined.

CHINA: 2♂2♀, Yunnan province, Dehong Autonomous Prefecture, Longchuan county. 28.iv.1981, Chikun Yang (CAU); 1♂, Yunnan province, Dehong Autonomous Prefecture, Ruili county. 1.v.1981, Fasheng Li (CAU); 1♂1♀, Hainan province, Wuzhishan city, Wuzhi hills. 16.v.2007, Yongjie Wang (CAU); 1♂, Hainan province, Baisha county, Yuanmen country, Hongxing village. 10.ix.2008, Xiushuai Yang (CAU).

#### Remarks.

This species was described by Banks (1939) based on one specimen from Hainan province. In this paper both male and female are described, including the genitalia, and the distribution records for this species in China are updated. This species is similar to *Zachobiella striata* but it can be easily distinguished by the different positions of 3ir1 in forewing.

## Supplementary Material

XML Treatment for Zachobiella yunanica

XML Treatment for Zachobiella submarginata

XML Treatment for Zachobiella striata

XML Treatment for Zachobiella hainanensis
